# Combining Understanding of Immunological Mechanisms and Genetic Variants Toward Development of Personalized Medicine for Psoriasis Patients

**DOI:** 10.3389/fgene.2019.00395

**Published:** 2019-05-03

**Authors:** Natalie Vivien Gunter, Bryan Ju Min Yap, Caroline Lin Lin Chua, Wei Hsum Yap

**Affiliations:** School of Biosciences, Taylor’s University, Subang Jaya, Malaysia

**Keywords:** psoriasis, autoimmune disorder, susceptibility loci, genome-wide association studies (GWAS), personalized medicine

## Abstract

Psoriasis is multifactorial disease with complex genetic predisposition. Recent advances in genetics and genomics analyses have provided many insights into the relationship between specific genetic predisposition and the immunopathological mechanisms driving psoriasis manifestation. Novel approaches which utilize array-based genotyping technologies such as genome-wide association studies and bioinformatics tools for transcriptomics analysis have identified single nucleotide polymorphisms, genes and pathways that are associated with psoriasis. The discovery of these psoriasis-associated susceptibility loci, autoimmune targets and altered signaling pathways have provided opportunities to bridge the gap of knowledge from sequence to consequence, allowing new therapeutic strategies for the treatment of psoriasis to be developed. Here, we discuss recent advances in the field by highlighting how immune functions associated with psoriasis susceptibility loci may contribute to disease pathogenesis in different populations. Understanding the genetic variations in psoriasis and how these may influence the immunological pathways to cause disease will contribute to the efforts in developing novel and targeted personalized therapies for psoriasis patients.

## Introduction

Psoriasis is a chronic, inflammatory skin disorder involving hyperproliferation of epidermal keratinocytes and neo-angiogenesis (Griffiths, 2003; Lowes et al., 2014; Brembilla et al., 2018). This autoimmune disorder is multifactorial and inflammation is known to play a major role in its development. Immunohistochemistry studies have showed that T cells are predominantly found in psoriatic lesions (Griffiths, 2003). Activated Th1 and Th17 T cells (CD4^+^ T cells) and CD8^+^ T cells, as well as increased levels of cytokines such as IL-17, IL-23, TNF-α and IL-27, have been directly implicated in psoriasis immunopathogenesis (Luger and Loser, 2018). Interestingly, recent studies have shown that different genetic variations in psoriatic patients are associated with distinct disease phenotypes (Puig et al., 2014). Recent advances in genomics such as genome-wide association studies (GWAS) and SNP arrays have revealed more than 40 psoriasis susceptibility loci (Mahil et al., 2015). Genes at these loci encode for proteins that are involved in skin barrier function andimmune cell signaling pathways (Puig et al., 2014). In this review, we will be discussing on the recent bioinformatics and experimental approaches which have led to successful discoveries of psoriasis-associated genes. In addition, we will also review on how these susceptibility loci are associated with dysregulated immune functions, which ultimately lead to the development of psoriasis, as summarized in Table 1. A better understanding of the immunopathogenic pathways in psoriasis hopefully can aid in development of novel personalized treatments for psoriasis patients.

**Table 1:** Relationship between psoriasis immunological mechanisms and susceptibility loci in different populations.

Classification	Immunological	Psoriasis	Associated	Relationship	References
	Mechanism/	Susceptibility	population		
	Target	loci			
Autoantigens	LL-37	*HLA-C^∗^06:02*	European, Chinese	LL-37 binds to HLA-C^∗^06:02. Complexes with self-DNA to enhance plasmacytoid dendritic cell production of IFN-γ.	Lande et al., 2007, 2014; Mabuchi and Hirayama, 2016
	ADAMTSL5	*HLA-C^∗^06:02*	European, Chinese	Complexes with HLA-C^∗^06:02 and is presented to Va3S1/Vb13S1 TCR in CD8+ cytotoxic T cell. Induces production of IL-17A and IFN-γ.	Arakawa et al., 2015; Fuentes-Duculan et al., 2017
Antigen presentation	B27	*HLA-B^∗^27*	European	Misfolded or aberrant HLA-B27 molecules present self-peptides to CD8^+^ T cells. Accumulation in endoplasmic reticulum stimulates ER stress response pathways and the release of pro-inflammatory cytokines.	Ruiz et al., 2012; Colbert et al., 2014; Eder et al., 2017
	ERAP1	*ERAP1* haplotype (rs27524, rs26653, rs30187, rs151823)	European, Romanian, Han Chinese, Chinese Uygur	Modulates processing of MHC class I molecule and the binding of antigenic peptides to MHC molecule.	Strange et al., 2010; Sun et al., 2010; Lysell et al., 2013; Tang et al., 2014; Kenna et al., 2015; Popa et al., 2016
	ERAP2	*ERAP2* haplotype (rs2248374, rs2910686)	European, Romanian	Modulates processing of MHC class I molecule and the binding of antigenic peptides to MHC molecule.	Popa et al., 2016
IL-20 Signaling	IL-20	*IL-20* HT GGA haplotype	North Indian	Upregulated IL-20 binds and induces STAT3 activation in keratinocytes, leading to an increase in cell proliferation and development of psoriatic lesions.	Lebre et al., 2012; Wani et al., 2018
IL-17/23 Signaling	p40 subunit of IL-12 and IL-23	*IL-12B* haplotype (A allele of rs3212227 and G allele of rs6887695)	European, Danish, Thai, Japanese	Increased expression of p40 subunit causes an increase of IFN-γ production, formation of IL-23 and biological activities of IL-12. Polarizes T cells to Th1 (by IL-12) and Th17 (by IL-23) lymphocytes.	Johnston et al., 2013; Zhu et al., 2013; Tamari et al., 2014; Zhao et al., 2016; Loft et al., 2018
	p19 subunit of IL-23	*IL-23A* haplotypes	European, Chinese	Promotes survival and expansion of Th17 lymphocytes and the subsequent release of IL-17, IL-22 and TNF-α. Results in dysregulated IL-23 signaling.	Nair et al., 2009; Bowes et al., 2011; Chen G. et al., 2016; Tsoi et al., 2017
	IL-17A	*TRAF3IP2*	European, Japanese	IL-17A and IL-17F interacts with IL-17R recruits TRAF3IP2, a positive signaling adaptor protein required for activation of NF-κB signal transduction and T-cell immune response.	Ellinghaus et al., 2010; Tamari et al., 2014; Tsoi et al., 2015, 2017
	PTTG1	*PTTG1* haplotype (rs2431697)	Han Chinese	The transcription factor coded by *PTTG1* regulates proliferation and differentiation of keratinocyte. Overexpression of the gene results in hyperproliferation and impaired keratinocyte differentiation, as well as overproduction of TNF-α.	Sun et al., 2010; Ishitsuka et al., 2013
NF-κB Signaling	IκB-zeta	*NFKBIZ*	European	Transcriptional regulator of NF-κB which binds to the p50 subunit of NF-κB. Also required for IL-17 dependent signaling. Defective *NFKBIZ* also affects development of Th17 lymphocytes.	Tsoi et al., 2015
	ABIN-1	*TNIP1* haplotype (rs2233278)	European, Japanese, Chinese	ABIN-1 regulates NF-κB cascades through linking of A20 to NEMO/IKKγ, resulting in the A20-mediated deubiquitination of NEMO/IKKγ and the inhibition of NF-κB. Defective protein results in dysregulated NF-κB signaling.	Bowes et al., 2011; Callahan et al., 2013; Tamari et al., 2014; Tsoi et al., 2017
Skin Barrier	LCE3C and LCE3B proteins	*LCE3C_LCE3B*-del	Chinese, Mongolian, European	Absence of LCE proteins involved in repair of skin barrier injury leads to abnormal keratinocyte proliferation and differentiation, forming an imperfect epidermal barrier.	de Cid et al., 2009; Hüffmeier et al., 2009; Zhang et al., 2009; Riveira-Munoz et al., 2011; Xu et al., 2011
	LCE3D protein	*LCE3D* haplotype (rs512208, rs4112788, rs4085613)	Han Chinese, Mongolian	Higher expression of *LCE3D* results in abnormal formation of cornified envelope and dysregulation of terminal epidermal differentiation.	Jackson et al., 2005; Zhang et al., 2009; Bergboer et al., 2011; Tang et al., 2014; Sun et al., 2018
Miscellaneous	AIM2	*AIM2* haplotype (rs2276405)	Han Chinese	AIM2 binds cytosolic dsDNA, forming an inflammasome and activates caspase-1 and subsequently IL-1β in keratinocytes.	Dombrowski et al., 2011; Zuo et al., 2015
	MGAT5	*MGAT5* haplotype	European, Spanish	MGAT5 is required for *N*-glycosylation of asparagine residues in HLA molecules. Deficiency of MGAT5 enzyme and its activity reduces threshold for T cell activation, increases risk of losing immune tolerance and promotes triggering of autoimmune diseases.	Demetriou et al., 2001; Aterido et al., 2016

## Tools for Genome-Wide Association Study of Psoriasis Patients

### Array-Based Technologies and Bioinformatics Analyses

Recent experimental approaches in GWAS have revealed various new psoriasis susceptibility loci (Chen W. et al., 2016; Visscher et al., 2017). The use of arrays such as Immunochip, a custom Illumina high-density SNP array, has allowed discovery of over 15 psoriasis susceptibility loci (Tsoi et al., 2012). This genotyping array has also been used to fine-map previously discovered immune-related susceptibility loci (Bowes et al., 2015). In addition, microarray data from Affymetrix microarray chips that were analyzed using gene set enrichment analysis (GSEA) led to the identification of 65 key genes associated with psoriasis (Chen W. et al., 2016). GSEA is a computational method that allows the study of gene expression levels between normal and disease states, and is especially helpful in detecting small changes in individual genes (Chen W. et al., 2016). Utilization of exome chips such as Illumina Human Exome Fine Mapping BeadChip allowed identification of coding variants (Zuo et al., 2015). Exome genotyping arrays has the ability to detect rare SNPs and is suitable for large-scale GWAS (Guo et al., 2014). The use of these chips and arrays also provided more insights into the etiology of psoriasis.

Existing array-based approaches in GWAS have also been combined with meta-analyses such as genotype imputation. Genotype imputation is described as ‘*in silico* genotyping,’ where computational analyses allow the evaluation of disease-associated genetic markers that have not been directly genotyped (Burdick et al., 2006; Li et al., 2009). For example, this method would allow individual genotypes to be determined by factoring in the distribution of the genotype between individuals such as in a pedigree or a specific population (Li et al., 2009). Therefore, this allows laboratory-based approaches such as genotyping using Illumina BeadChips or real-time polymerase chain reaction (RT-PCR) to be supplemented with this computational method, where partial information of each gene can be combined and incorporated in the association analysis (Li et al., 2009; Aterido et al., 2016; Popa et al., 2016). Thus, the association hits first discovered through the array-based approach can be improved by subsequent association analysis on imputed variants (Wang and Chatterjee, 2017). In addition to that, imputation also increases the power of detection in genotypic variability-based GWAS (vGWAS) which studies non-additive loci and their effects on disease phenotypes (Wei et al., 2018).

It is also important to note that many single-marker GWAS only consider genes individually without assessing the combinatory effect of multiple causal variants or the biological consequences (Aterido et al., 2016). Genome-wide pathway analysis allows integration of such genetic and biological aspects to test functionally-related genes associated with a complex trait. For example, *CXCR4* gene which contributes to the pathways driven by *IL12B* gene was not previously associated with psoriasis susceptibility in single-marker GWAS but was being implicated as part of the central mechanisms of disease pathophysiology using genome-wide pathway analysis (Aterido et al., 2016). Thus, multi-genotype combination analysis enables a more elaborate study of psoriasis pathogenesis and inheritance patterns (Dou et al., 2017). PLINK software is used in this analysis to identify the association of psoriasis risk with genetic pathways and between genes in relation to psoriasis susceptibility (Bowes et al., 2015; Aterido et al., 2016). Besides that, a new algorithm, minimum distance-based enrichment analysis for genetic association (MEAGA), was recently developed to relate GWAS data to biological functions and pathways. Using this algorithm, overlapping of genes of enriched functions can be identified, linking functional and regulatory networks with psoriasis immunopathogenesis (Tsoi et al., 2017). In contrast to functional variants, non-coding variants, on the other hand, have been studied for their regulatory functions using HaploReg (v2) and expression quantitative trait locus (eQTL) databases (Yin et al., 2015). Another method of analysis used in association studies is conditional analysis, which is used to identify secondary association signals at a locus. This means that analysis by conditioning to the primary associated SNP allows testing for other significantly associated SNPs, which is useful for analyzing loci with multiple associated variants (Yang et al., 2012).

### Sequencing-Based Technologies

Despite the ability of SNP arrays in identifying disease-associated genes, further molecular experiments usually needs to be carried out to confirm the implication of having these genes (Chen W. et al., 2016). Another suggested approach for GWAS analysis is through whole genome sequencing (WGS). Using this method, every variant can be directly identified through genotyping and allows concurrent discovery and fine mapping of causal variants (Wang and Chatterjee, 2017). A related approach is the whole exome sequencing (WES) which allows all protein-coding regions to be sequenced (Petersen et al., 2017). For example, using exome and targeted sequencing in a study on Chinese population, numerous missense single-nucleotide variants including *LCE3D, ERAP1*, and *CARD14* were identified to be associated with the disease (Tang et al., 2014). Rarer variants in *IL23R*, *GJB2, TARBP1*, and *FUT2* were also identified and suggested associations (Tang et al., 2014). These techniques are shown to be unbiased and provide data on variant frequencies in different populations (Petersen et al., 2017; Wu et al., 2017). A remarkable example was demonstrated by Zhou et al. (2016) where the entire MHC region in the Han Chinese population was sequenced, allowing the construction of a Han-MHC reference panel which provides a summary of polymorphisms including SNPs and indels in the region. Besides discovering several new psoriasis susceptibility loci within the MHC region, development of this population-specific reference panel allowed comparison with other populations whereby a significant difference in HLA allele frequencies was discovered between Han Chinese and European populations (Zhou et al., 2016). Large-scale sequencing approaches however have not been utilized in GWAS as it is cost-ineffective and requires an extremely large sample size for discovery as opposed to array-based approaches (Petersen et al., 2017; Wang and Chatterjee, 2017).

## Genetic Variants and Their Association With Immuno-Pathological Mechanisms in Psoriasis

### Autoantigens and Antigen Presentation

#### Psoriasis Pathogenesis

The presence of autoantigens in psoriasis patients is a well-known factor that contributes to disease pathogenesis. Psoriasis patients may overexpress certain self-antigens which can be taken up by antigen presenting cells (APCs). Following this, the APCs may present the self-antigens on their MHC molecules and subsequently activate T cells via T cell receptors, triggering immune activation and attack on self-tissues. A study by Lande et al. (2014) found that self-reactivity to LL-37, an antimicrobial peptide, was found in two-thirds of patients with moderate-to-severe plaque psoriasis. LL-37 is produced by multiple cell types in the skin, such as keratinocytes and APCs, as a response to skin injury or bacterial layer. LL-37 then complexes with extracellular nucleic acids released during inflammation to activate myeloid dendritic cells (mDC) and plasmacytoid dendritic cells (pDC). These activated APCs release IL-23, which in turn stimulates Th17 T cells to produce IL-17A (Lande et al., 2014;Kim and Krueger, 2015; Hawkes et al., 2017b).

#### Susceptibility Loci in Psoriasis

In psoriasis patients, sustained stimulation of the IL-23-producing APCs by the abundance of LL-37-nucleic acid complexes contributes to the development of psoriatic inflammation. A few cohort studies of psoriatic patients from European and Chinese lineages have revealed that psoriasis and psoriatic arthritis patients have the human leukocyte antigen (HLA)-class I allele *HLA-C^∗^06:02* susceptibility locus, which is linked to the presentation of LL-37 autoantigen (Lande et al., 2007, 2014; Mabuchi and Hirayama, 2016; Fuentes-Duculan et al., 2017). LL-37 peptide can bind to HLA-Cw6^∗^02 to form a complex, which is presented by dendritic cells to CD4^+^ and CD8^+^ T cells. The specific recognition of the LL-37-HLA-Cw6^∗^02 complex induces proliferation of these reactive T cells, which is not seen in stimulation by other antimicrobial peptides. The proliferation, along with production of IL-17 and IL-22, correlates with PASI, where 15 out of 20 (75%) of patients PASI > 10 responded to LL-37 (Lande et al., 2014). ADAMTSL5, on the other hand, is a protein of the ADAMTS superfamily of metalloproteases that is proposed to play a role in microfibril formation and extracellular matrix regulation. This autoantigen has been associated with the melanocyte-derived model of psoriasis immunopathogenesis (Hawkes et al., 2017b). In psoriasis patients, their melanocytes were shown to express increased levels of ADAMTSL5 (Arakawa et al., 2015). These autoantigens are then presented by the melanocytes on their MHC class II molecules to epidermal CD8^+^ T cells, which are activated to release IL-17A. IL-17A may subsequently induce the production of chemokines such as CXCL1, which promotes melanocyte growth and therefore, ADAMTSL5 expansion. Recent findings also showed that ADAMTSL5 can be overexpressed in keratinocytes, thus melanocytes may not be the only autoimmune targets in psoriasis (Fuentes-Duculan et al., 2017; Hawkes et al., 2017a). Similarly, psoriasis patients with *HLA-C^∗^06:02* susceptibility locus can also present ADAMTSL5 peptide as an autoantigen. The peptides are presented by *HLA-C^∗^06:02*-positive melanocytes to CD8^+^ T cells via Vα3S1/Vβ13S1 T cell receptor (TCR), activating production of IL-17 and IFN-γ (Arakawa et al., 2015; Bonifacio et al., 2016; Fuentes-Duculan et al., 2017).

Another psoriasis susceptibility locus, *HLA-B^∗^27*, encoding the MHC class I HLA-B27 molecule, was strongly associated with psoriatic arthritis in European populations and is suggested to be the strongest genetic marker for this disease (Ruiz et al., 2012; Eder et al., 2017). Several mechanisms linking this susceptibility locus to psoriatic arthritis development have been proposed. One mechanism is the ability of B^∗^27 to present arthritogenic peptide to CD8^+^ T cells. Another suggested mechanism is the accumulation of B^∗^27 heavy chain within the endoplasmic reticulum (ER), which subsequently promotes the release of pro-inflammatory cytokines (Colbert et al., 2014; Queiro et al., 2015; Bowes et al., 2017). In addition to MHC class I genes, endoplasmic reticulum aminopeptidase (*ERAP1* and *ERAP2*) gene variations have been associated with psoriatic arthritis in the Romanian population (Popa et al., 2016). These aminopeptidases are found within the ER and function to cleave proteins into smaller peptides before they are presented on MHC class I molecules. Interestingly, it was also reported that the *ERAP1* gene haplotypes (rs30187) are only associated with psoriatic arthritis in patients with the *HLA-B^∗^27* susceptibility locus, while *ERAP1* haplotypes (rs27524, rs26653, and rs30187) also affects psoriasis susceptibility, but only in individuals with the *HLA-C* risk allele (Strange et al., 2010; Lysell et al., 2013; Kenna et al., 2015; Popa et al., 2016). Besides that, another *ERAP1* haplotype (rs151823) has been identified, demonstrating similar association with the *HLA-C* and type 1 psoriasis in Chinese populations (Sun et al., 2010; Tang et al., 2014). In contrast, *ERAP2* gene haplotypes (rs2248374, rs2910686) are associated with disease in patients without the *HLA-B^∗^27* susceptibility locus (Popa et al., 2016).

### Cytokine Signaling Pathways

#### Psoriasis Pathogenesis

**Figure 1:**
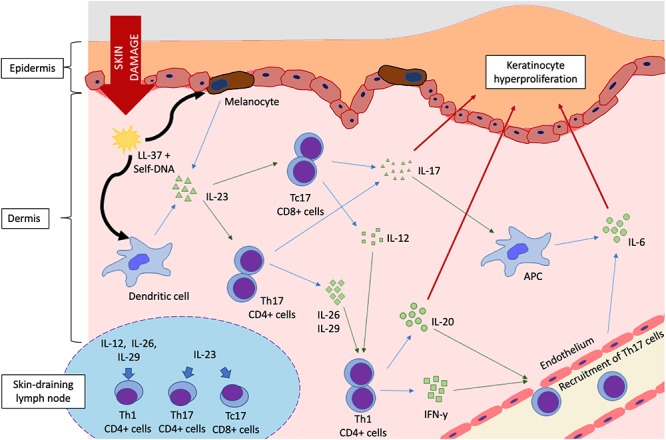
Cytokine signaling pathways in the pathogenesis of psoriasis. Damage to the epidermal lining triggers release of antimicrobial peptides (AMP) such as LL-37, which complexes with self-DNA released from cellular membrane rupture. DNA-LL-37 complexes are autoantigens of psoriasis, which are taken up by melanocytes and dendritic cells, resulting in IL-23 production. The IL-23/IL-17 axis is activated in a feedforward loop that favors keratinocyte proliferation, eventually forming a psoriatic plaque. Schematic of lymph node (bottom left) shows the polarization of naïve T cells into Th1, Th17, and Tc17 cells by cytokine stimulation. (Key: Black arrows, uptake; Blue arrows, cytokine production; Green arrows, stimulation or activation; Red arrows, effect on epidermal keratinocytes).

Therapies targeting IL-23 and/or IL-17 have shown strong efficacy in the management of psoriasis, affirming the central role of these cytokines in psoriatic inflammation (Prinz, 2017) (Figure 1). IL-23 is produced abundantly by keratinocytes and activated APCs, such as Langerhans cells and dendritic cells. Upon binding of IL-23 to its receptor (IL-23R on naïve T cells), the JAK2/Tyk downstream signaling pathway is activated, resulting in the phosphorylation of STAT3. In the presence of IL-23, the number of pathogenic T17 cells [Tc17 (CD8+) and Th17 (CD4+)] are increased dramatically, producing large amounts of IL-17. Other cell types, such as αβ T cells, dermal γδ T cells, neutrophils and mast cell, are also capable of producing IL-17 in response to IL-23 stimulation (Blauvelt and Chiricozzi, 2018). Other cytokines produced by T17 cells, including IL-26 and IL-29, activate STAT1 to upregulate chemokines and promote recruitment of Th1 cells (Chan et al., 2018).

Keratinocytes, endothelial cells and cells of innate immunity are targets of IL-17. IL-17 can upregulate inflammatory gene expression and activate psoriasis-associated genes in epidermal keratinocytes upon binding to the IL-17R family of receptors (Amatya et al., 2017). IL-17 can also act synergistically with TNF-α to further upregulate these genes (Blauvelt and Chiricozzi, 2018; Chan et al., 2018). The IL-17 family of interleukins consist of IL-17A, B, C, D, E, and F. In psoriasis, IL-17A is known as the principal effector molecule that triggers inflammation, with the closely related IL-17F playing a similarly significant role. IL-17 can directly act on keratinocytes, inducing the production of IL-19 and IL-22 that promote hyperproliferation and dysregulated differentiation of epidermal keratinocytes (Chan et al., 2018). Increased levels of IL-17 can significantly upregulate the production of antimicrobial peptides in keratinocytes, IL-6, and ICAM-1 in endothelial cells to promote inflammation, and pro-inflammatory cytokines such as IL-6 in APCs (Blauvelt and Chiricozzi, 2018). ICAM-1 is a vascular adhesion molecule expressed by endothelial cells, lymphocytes and keratinocytes, and is essential for T cell migration to the skin (Bressan et al., 2018). IL-6, IL-12, IL-19, and IL-22 are associated with keratinocyte proliferation (Grossman et al., 1989; Shaker et al., 2006; Stenderup et al., 2007; Torti and Feldman, 2007). IL-17 was also shown to promote the expression of IL-23 and STAT3 in a study involving reconstructed human epidermal model (Chiricozzi et al., 2014). These events induce a positive feedback mechanism to further increases the production of both IL-23 and IL-17, creating a cycle that amplifies the inflammation and accelerates the development of psoriasis.

Another cytokine, IL-12, was found to be highly expressed in psoriatic lesions. IL-12 is a pro-Th1 heterodimeric cytokine composed of covalently-linked p40 and p35 subunits (Shaker et al., 2006). The p40 subunit of IL-12 and IL-23 is thought to be the main regulator in psoriasis and psoriatic arthritis, being associated with the formation of these interleukins and polarization of Th1 and Th17 lymphocytes respectively (Johnston et al., 2013; Zhao et al., 2016). However, IL-12 only marginally induced production of IL-17 by Th17 (Aggarwal et al., 2003). The production of IL-12 by CD4^+^ Th1 and CD8^+^ cytotoxic T cells along with IFN-γ and TNF-α in a pattern known as the type I cytokine pattern contributes to pathogenesis of psoriasis. IL-12 stimulates T cells to produce IFN-γ, which induces keratinocytes to express ICAM-1 and HLA-DR. This facilitates epidermal infiltration of T cells, which subsequently activates keratinocytes, leading to their proliferation and development of plaque psoriasis (Shaker et al., 2006; Torti and Feldman, 2007). IL-20, a pro-inflammatory cytokine that is associated with angiogenesis and chemotaxis of leukocytes, has also been found at increased levels in psoriatic lesions. It is expressed by epidermal keratinocytes together with the IL-20 receptor (IL-20R), which suggests an autocrine signaling mechanism may be in place to maintain the inflammation in psoriasis. A previous study demonstrated that blocking IL-20 signaling in immunocompromised mice that were grafted with psoriatic plaques was able to inhibit the development of disease, proving its role in the maintenance of psoriatic inflammation (Stenderup et al., 2009). Another cytokine, IL-19, is known to contribute to psoriasis by inducing production of antimicrobial peptides, IL-23p19 peptides and Th17-attracting chemokines. IL-19 is produced by keratinocytes in response to IL-17, showing a feedforward loop that increases IL-17 by recruitment of Th17 cells (Witte et al., 2014).

Previous studies have identified mutations in a gene known as *caspase recruitment domain family member 14 (CARD14)* gene as a mediator in the immunopathogenesis of psoriasis. The gene is expressed mainly in epidermal keratinocytes and encodes for the CARD14 protein, also known as CARMA2. CARD14/CARMA2 is a scaffold protein that can activate NF-κB, a transcription factor that regulates multiple genes including those responsible for the production of pro-inflammatory cytokines implicated in the pathogenesis of psoriasis. A study reported that CARMA2 mutation which involves the deletion of glutamic acid at position 138 (CARMA2Δ138) resulted in spontaneous development of psoriasis in C67BL/6J mice (Mellett et al., 2018). A single copy of the gain-of-function mutation is sufficient to cause pathology. In addition, IL-23 and imiquimod treatments to induce psoriasis were shown to be unsuccessful in CARD14/CARMA2-deficient mice (Tanaka et al., 2017).

Few studies have sought to better understand the underlying mechanisms linking mutations in *CARD14* gene to psoriasis development. Gain-of-function genetic mutations (*CARD14*^E138A^ and *CARD14*^G117S^) were reported to cause constitutive activation of CARD14 signaling, by promoting their interaction with BCL10 and MALT1 proteins (Howes et al., 2016). The resulting complex is known as the CARMA-BCL10-MALT1 (CBM) complex, which can trigger NF-κB-activation, ultimately leading to cytokine production and epidermal keratinocytosis (Wang et al., 2018; Zotti et al., 2018). Mice with *CARD14* genetic mutations (*CARD14*^E138A/+^ and *CARD14*^DQ136/+^) were shown to spontaneously develop psoriasis-like disease due to hyper-activation of NF-κB and enhanced activation of IL-17A signaling in keratinocytes (Wang et al., 2018).

#### Susceptibility Loci in Psoriasis

Several psoriasis susceptibility loci have been associated with cytokine signaling pathways such as IL-20, IL-17/23 and NF-κB signaling pathways. Recent studies reported *IL-20* HT GGA haplotype as the susceptibility loci for both psoriasis and psoriatic arthritis in North Indian population, where patients with this haplotype had increased IL-20 levels (Lebre et al., 2012; Wani et al., 2018). The *IL-12B* risk haplotype (A allele of rs3212227 and G allele of rs6887695) has been identified in European, Danish, and Asian populations, specifically the Thai and Japanese populations (Johnston et al., 2013; Zhu et al., 2013;Guo et al., 2014; Tamari et al., 2014; Loft et al., 2018). Meanwhile, several SNPs in the *IL23A* gene which code for the p19 subunit of IL-23 identified in European and Chinese populations have been associated with psoriasis susceptibility and immunopathogenesis via IL-23 signaling (Nair et al., 2009; Bowes et al., 2011; Li et al., 2016; Tsoi et al., 2017). Another susceptibility locus, though with lower frequency, was identified in the *IL23R* gene with haplotypes rs11209026 and rs7530511 associated with European populations, and haplotype rs3762318 for Chinese populations (Tang et al., 2014). In addition, *TRAF3IP2* has been identified as a susceptibility locus in European and Japanese populations for both psoriasis and psoriatic arthritis (Ellinghaus et al., 2010; Tamari et al., 2014; Tsoi et al., 2015, 2017). *TRAF3IP2* encodes for an adaptor protein that is involved in IL-17 and NF-κB signaling, where binding of IL-17A and IL-17F to IL-17R leads to TRAF3IP2 recruitment, and subsequently activates NF-κB pathway and inflammatory response (Ellinghaus et al., 2010). Another psoriasis susceptibility locus, *NFKBIZ*, has been identified in European populations (Tsoi et al., 2015). The gene encodes for transcriptional regulator IκB-zeta, which plays an important role in the regulation of IL-17 signaling and development of Th17 cells (Tsoi et al., 2015). Other loci involved in modulation of NF-κB pathway in psoriasis and psoriatic arthritis includes the susceptibility loci *TNIP1* gene haplotype (rs2233278), which was identified in Japanese, European, and Chinese populations (Bowes et al., 2011; Callahan et al., 2013; Tamari et al., 2014; Tsoi et al., 2017). The A20-binding protein, ABIN-1 coded by *TNIP1* controls and restricts several NF-κB cascades through interaction with A20 to NEMO/IKKγ (Callahan et al., 2013; Tamari et al., 2014). Besides that, *CARD14* gene has also been discovered to be a susceptibility locus with the common risk haplotype rs1165075 identified in European, Spanish, and Chinese populations (Tsoi et al., 2012; González-Lara et al., 2013; Tang et al., 2014). Mutations in this gene result in a gain-of-function, causing constitutive activation of the NF-κB pathway and as a result increases the production of pro-inflammatory cytokines (Tsoi et al., 2012; González-Lara et al., 2013).

A stop-gained variant at psoriasis susceptibility locus *AIM2* (rs2276405) which codes for a cytosolic double-stranded DNA receptor has been identified in Han Chinese population (Zuo et al., 2015). Increased cytosolic DNA and AIM2 expression in keratinocytes results in the formation of inflammasomes, which trigger caspase-1 activation and subsequent pro-inflammatory IL-1β release (Dombrowski et al., 2011; Zuo et al., 2015). Interestingly, while LL-37 serves as an autoantigen in psoriasis immunopathogenesis, studies suggest that it can also inhibit AIM2-mediated inflammasome formation (Dombrowski et al., 2011). Meanwhile, *MGAT5* gene was recently shown to be associated with psoriasis susceptibility in Spanish and European populations (Aterido et al., 2016). MGAT5 enzyme deficiency reduces the threshold required for the activation of T cells, thus increases the risk of losing immune tolerance and promotes susceptibility to autoimmune diseases such as psoriasis (Demetriou et al., 2001; Aterido et al., 2016).

Another identified susceptibility loci *PTTG1* haplotype (rs2431697) highlights population-dependent effects, whereby it was associated with psoriasis in Han Chinese populations but not in European populations (Sun et al., 2010). This gene codes for the transcription factor involved in regulating the proliferation and differentiation of keratinocytes and was found to be overexpressed in psoriasis which subsequently leads to overproduction of TNF-α and the resultant inflammation (Ishitsuka et al., 2013). Sun et al. (2010) also reported other psoriasis susceptibility loci such as *CSMD1*, *GJB2, SERPINB8* and *ZNF816A* in Han Chinese population.

### Skin Barrier

#### Psoriasis Pathogenesis

Human skin is equipped with barrier function to prevent entry and invasion of pathogens. It is composed of physical barrier, permeability barrier, and innate and adaptive barriers. The permeability barrier lies within the stratum corneum layer and is dependent on corneocytes and the lipid-rich matrix surrounding the cells (Sano, 2015). Stratum corneum is formed through terminal differentiation of epithelial keratinocytes. It is composed of various lipids and proteins, including members of the late cornified envelope (LCE) protein family (Niehues et al., 2016). The LCE gene cluster is located on chromosome 1q21, where it encodes for 18 proteins whose functions are largely unknown. It is hypothesized that the LCE genes encode for structural proteins are involved in the repair of skin barrier and play an important role in cornified epithelial differentiation (Jackson et al., 2005; Sano, 2015). In particular, LCE3B and 3C proteins are known to be crucial in promoting the recovery of the skin barrier. In the normal skin, LCE3B and 3C proteins are usually expressed at low to negligible levels, but their expression can be induced following mechanical stripping of the cornified epithelial layer (de Cid et al., 2009). However, in individuals without these genes, the epidermal barrier cannot be properly repaired due to abnormal keratinocyte differentiation and proliferation (Zhang et al., 2009; Xu et al., 2011). The compromised skin barrier enables easier penetration of exogenous agents, which may then activate host immune responses in the skin and promote psoriatic development (de Cid et al., 2009; Coto et al., 2011). This mechanism of immune activation leading to psoriasis is especially common in patients who are *HLA-Cw6* positive (de Cid et al., 2009).

#### Susceptibility Loci in Psoriasis

In the recent years, discovery of a biallelic *LCE3C_LCE3B-del* copy number variant has been identified and was linked to psoriasis susceptibility (de Cid et al., 2009; Riveira-Munoz et al., 2011). This 32.2-kb gene deletion removes functioning genes of *LCE3C* and *LCE3B* in the LCE cluster on chromosome 1q21.3 (Riveira-Munoz et al., 2011). In addition to gene deletion, various studies have characterized several associations between this LCE susceptibility loci and other risk factors in different populations. For example, epistatic effect with *HLA-Cw6* allele was reported in Dutch and US Michigan populations (de Cid et al., 2009; Riveira-Munoz et al., 2011). This association with the *HLA-Cw6* status, however, was not marked in other studied populations including Chinese, Mongolian and other European ancestries (de Cid et al., 2009; Hüffmeier et al., 2010; Riveira-Munoz et al., 2011; Xu et al., 2011). It was hypothesized that these distinct findings in different populations may be attributed to variations in genetic backgrounds, as well as environmental factors (Xu et al., 2011). A few studies have also investigated if the *LCE3C_LCE3B-del* gene loci are specifically associated only with the development of skin-related psoriasis vulgaris, or with psoriatic arthritis as well. Two contrasting findings were obtained in different populations; there was no association between this gene loci and psoriatic arthritis in the German population (Hüffmeier et al., 2009), while other studies done on the British population found an association between this loci and psoriatic arthritis (Bowes et al., 2010; Zhang et al., 2016).

Besides that, another susceptibility locus was identified within the LCE cluster, specifically haplotypes of *LCE3D* gene (rs512208, rs4112788, rs4085613) in Han Chinese and Mongolian populations (Zhang et al., 2009; Tang et al., 2014; Sun et al., 2018). The late envelope protein 16 which is coded by this gene is involved in the formation of the cornified envelope as well as the regulation of terminal epidermal differentiation (Jackson et al., 2005). Studies reveal a higher expression of *LCE3D* in psoriatic samples and is thus hypothesized to contribute to the formation of psoriatic lesions (Bergboer et al., 2011).

## Genetic Variants and Personalized Medicine for Psoriasis

Identifying the causal alleles within the refined association signals will help to guide the development of targeted treatments. Pathogenic insights obtained from large-scale GWAS studies have identified IL-23 and IL-17 as key disease drivers that can be targeted by various classes of therapeutics. It informed the development of ustekinumab, a drug which targets the p40 subunit shared by IL-12 and IL-23 (SNPs in *IL12B* which encodes p40 are associated with psoriasis susceptibility). The p40 unit shared by IL-12 and IL-23 is an attractive therapeutic target as it influences two important effector cytokines, IFNγ and IL-17. Meanwhile, the development of IL-23A and IL-17 inhibitors were informed by GWAS analysis which led to the development of IL-17 blockers such as Secukinumab (Cosentyx), ixekizumab (Taltz) and broadalumab (Kyntheum) and IL-23 inhibitors including Guselkumab (Tremfya), and tildrakizumab. There are no risk alleles in *IL17A*, but psoriasis-associated SNPs have been identified in *TRAF3IP2*, which encodes an IL-17 receptor adaptor while SNPs in *IL23A*, which encodes p19, are associated with psoriasis susceptibility.

The current treatments for psoriasis however are limited by inter-individual variation in efficacy. Advances in GWAS allow researchers to make further associations between genetic variants of genomic loci and their corresponding phenotypic differences, which assist in risk prediction for targeted prevention or intervention strategies and provide discovery pipeline for new drug. In clinical trials, PASI-75 was achieved in more than 60% of ustekinumab-treated psoriasis patients at 12 weeks. Interestingly, current associations between the *HLA-Cw^∗^06* genotype and response to ustekinumab have shown conflicting findings. While a retrospective study of 255 patients have shown 71.7% of *Cw^∗^06*-positive patients reached PASI 50 at week 4 compared with 35.2% of those who were *Cw^∗^06*-negative (Talamonti et al., 2017), a differential response was also reported in a study of 332 patients in which 62% of *Cw^∗^06*-positive patients vs. 48% of *Cw^∗^06*- negative patients reached PASI 50 after 4 weeks of therapy (Li et al., 2016). In contrast, some even found no association of *Cw^∗^06* genotype with response to ustekinumab treatment in 69 patients with psoriasis treated with ustekinumab (Prieto-Perez et al., 2017). These pharmacogenetic studies have identified the effects of variants in specific genes that are associated with clinical response to treatments, which can be used to inform patient care and reduce drug-related costs.

Collaborative efforts from various stakeholders may be able to assist in detailed assessment of GWAS data. Recently, the Psoriasis Stratification to Optimize Relevant Therapy (PSORT) consortium was formed to improve understanding of the determinants of responses to biologic therapies for psoriasis^1^. It uses the large-scale United Kingdom-based clinical data resource called British Association of Dermatologists’ Biologic Interventions Registry and integrates this with genetic, immune, and transcriptomic data for patients treated with biologics. Data gathered from large scale consortium studies will provide insights for personalized medicine in psoriasis patients whereby individual genetic profiles can be used to define prognosis, including the disease subtype, response to specific medications, and prediction of potential adverse drugs reactions.

## Conclusion

Although GWAS contributed insight into the utility of the genotype biomarker to treatment selection, adequately powered prospective studies will be required before clinical application of pharmacogenomics can become a reality. Genotype and phenotype assessment should be facilitated by the availability of detailed molecular analyses and data integration. Careful assessment of prospective GWAS data is essential to integrate findings into the clinical decision-making process, and thereby optimizing the treatment of patients with psoriasis in the future.

## Author Contributions

BY, NG, and WY performed the project. CC provided vital guidance and insight to the work. WY and CC conceptualized the project.

## Conflict of Interest Statement

The authors declare that the research was conducted in the absence of any commercial or financial relationships that could be construed as a potential conflict of interest.

## Footnotes

1 www.psort.org.uk
